# New Insights into Glomerular Parietal Epithelial Cell Activation and Its Signaling Pathways in Glomerular Diseases

**DOI:** 10.1155/2015/318935

**Published:** 2015-03-19

**Authors:** Hua Su, Shan Chen, Fang-Fang He, Yu-Mei Wang, Philip Bondzie, Chun Zhang

**Affiliations:** ^1^Department of Nephrology, Union Hospital, Tongji Medical College, Huazhong University of Science and Technology, Wuhan, Hubei 430022, China; ^2^Department of Pathology and Lab Medicine, Boston University School of Medicine, Boston, MA 02118, USA

## Abstract

The glomerular parietal epithelial cells (PECs) have aroused an increasing attention recently. The proliferation of PECs is the main feature of crescentic glomerulonephritis; besides that, in the past decade, PEC activation has been identified in several types of noninflammatory glomerulonephropathies, such as focal segmental glomerulosclerosis, diabetic glomerulopathy, and membranous nephropathy. The pathogenesis of PEC activation is poorly understood; however, a few studies delicately elucidate the potential mechanisms and signaling pathways implicated in these processes. In this review we will focus on the latest observations and concepts about PEC activation in glomerular diseases and the newest identified signaling pathways in PEC activation.

## 1. Introduction 

The glomerulus comprises four types of intrinsic cells including endothelial cells, mesangial cells, podocytes, and parietal epithelial cells (PECs). There are numerous studies focused on the biological functions and pathogenic roles of the first three cells, whereas PEC, which lines along Bowman's capsule, until recently has aroused scientific interest leading to the exploration of its physiological and pathological effects especially in several forms of glomerular diseases, such as crescentic glomerulonephritis (CGN), focal segmental glomerulosclerosis (FSGS), and diabetic nephropathy (DN).

Quiescent PECs are very flat and inconspicuous; their cell body size ranges from 0.1 to 0.3 *μ*m in thickness, increasing to 2.0~3.5 *μ*m at the nucleus. Transmission electron microscopic studies indicate PECs have a “labyrinth-like” outlook with junctions present between adjacent PECs. At the glomerular urinary pole, PECs develop junctions with proximal tubular cells, while, in the vascular pole, PECs transit into podocytes [1].

Comparing to other kidney resident cells, there are a few universally accepted concepts about the physiologic functions of PECs. Several best evidence-based researches suggest they act as a selective permeability barrier to urinary filtrate [2]. Moreover, in albumin overload state, PECs can uptake albumin likely by endocytosis which eventually leads to the injury of PECs [3]. In addition, PECs may serve mechanosensing and contractile functions through their primary cilia which are constantly exposed to the urine flow from the glomerular filtrate [4].

Recently Shankland et al. [5] published an elegant review about the emerging concepts of PECs. In this review we summarize PECs activation in several kinds of glomerular diseases, particularly the signaling pathways implicated in PECs activation.

## 2. Parietal Epithelial Cell (PEC) Activation in Glomerular Disease

Currently, no well-defined glomerular disease is predominantly caused by abnormalities arising in PECs, unlike other glomerular diseases that occur mainly due to the injury of certain intrinsic glomerular cells, such as podocytes (minimal change disease and focal segmental glomerulosclerosis), mesangial cells (IgA nephropathy), and glomerular endothelial cells (thrombotic microangiopathies and pauci-immune glomerulonephritis) [6]. Nonetheless, emerging data suggest that PECs are directly involved in the pathogenesis of certain glomerular disease entities, which are featured by increased cellular activity of PECs. Activated PECs have enlarged nuclei and increased cuboidal cytoplasm, and occasionally cytoplasma vacuolation and protein resorption droplets are observed [2, 7–9]. Besides morphological alterations, CD44, phosphorylated extracellular signal regulated kinase, and several molecules are considered to be the specific markers for activated PECs [7]. After activation, the biological properties of PECs are changed, presenting as increased proliferation, migration, or extracellular matrix production [1].

The precise role of PECs in disease states remains unclear; it might be potentially detrimental or beneficial in glomerular diseases. On the one hand, the overgrown PECs could obstruct the urine flow and release chemokines and cytokines, which could impair the function of the affected glomeruli. On the other hand, several studies have suggested that PECs could perhaps migrate from Bowman's capsule to the capillary tuft and differentiate into podocytes in response to injury [10, 11]. In the latter circumstance, PECs serve a reparative and regenerative role when podocytes are lost. Several studies have shown the involvement of the activation of PECs in various glomerular diseases in humans and mouse models (Table 1).

### 2.1. PEC Activation in Crescentic Glomerulonephritis

CGN is the best-characterized disease in which PECs are the major culprits. Cellular crescent is the typical morphological change observed in CGN. It is defined as the multilayered accumulations of PECs and other cell types within Bowman's space. Consequently it occludes the urinary outlet and the flow of the primary urine, and later the implicated nephron is impaired.

No consensus has been reached with regard to the cell types inside the classic crescent, due to the fact that crescentic lesions often stain positive for both PEC and podocyte markers [9, 12]. However, at least three cell types contribute to the cellular components of classic crescents, either individually or in combination. Firstly, numerous studies have shown that PECs are the predominant cells in cellular crescents. Ultrastructural studies performed in the 1970s showed that crescents were largely composed of PECs and to a lesser extent podocytes [13]. In addition immunohistological staining confirmed that cellular crescents mainly expressed PECs markers, for example, CD24, glycCD133, and claudin-1 in humans and cytokeratin and UCH-L1 in rats [14]. More importantly, genetic cell-fate tracking studies elegantly demonstrated that PECs were the predominant source of cellular crescents in mice [9]. Secondly, under specific circumstances, podocytes contribute to cellular crescent formation to a much less extent in human diseases [12, 15] and animal model [16, 17]. For the latter, it is especially dependent on the experimental setting, such as the use of different anti-GBM serum to induce disease. Lastly, infiltrating macrophages have also been implicated as the potential origin of cellular crescents [18]. All in all, PECs are the predominant components of cellular crescents although they are likely to have multicellular origin.

PECs present in cellular crescents undergo epithelial-to-mesenchymal transition (EMT) potentially due to the deposition of extracellular matrix (ECM) proteins. The accumulation of ECM proteins results in the development of fibrocellular crescents. EMT in PECs is characterized by the loss of epithelial polarity and increased extracellular matrix synthesis [19], which eventually generates classical honeycomb-like lesions.

Undoubtedly, PECs are the main players in CGN, but they do not etiologically account for this pathogenic abnormality. Rather, glomerular tuft necrosis caused by vascular injury is the driving force for PECs proliferation, crescent formation, and consequent renal impairment. PECs usually settle in an environment without plasma exposure; nonetheless, when the glomerular capillary wall is ruptured the concentration of plasma within Bowman's space is increased around 20–40%. Amounting evidence proposes that plasma leakage initiates PECs activation and crescent development. The components from plasma markedly promote murine and human PECs hyperplasia in culture [20]. The plasma gradients that account for PECs activation have not been fully identified, but to date there is consistent data which shows fibrinogen, a member of the activated coagulation cascade, to be a driver for PECs activation. Accordingly, in several rodent models crescents formation was prevented in the absence of fibrinogen [20, 21].

### 2.2. PEC Activation in Noncrescentic Glomerulonephritis

CGN is frequently accompanied with inflammatory and necrotizing processes; thus, it is believed that inflammatory component is the main driving force for crescent formation. However, Sicking et al. [22] demonstrated that partial transgenic depletion of PECs was sufficient to trigger the activation of the remaining PECs population; eventually, these cells filled Bowman's capsule and formed cellular crescents. This suggests that cellular crescents might be developed without primary inflammatory stimulus. Consistently, crescent formation was noted in some patients with noninflammatory glomerular diseases including FSGS, DN, and membranous nephropathy (MN).

#### 2.2.1. PEC Activation in Focal Segmental Glomerulosclerosis

Collapsing variant of focal segmental glomerulosclerosis (cFSGS) is morphologically featured by segmental to global collapse of the capillary tuft with dramatic hyperplasia within Bowman's capsule, often termed pseudocrescents.

However, the origin of the proliferating cells in cFSGS is a debating issue. The traditional opinion believes that the overgrown cells stem from podocytes without podocyte markers due to the fact that they are dedifferentiated or dysregulated podocytes and they reentered the cell cycle to mitosis [23–25]. However in human HIV and pamidronate-associated cFSGS, the PECs markers were obviously expressed in proliferating epithelial cells within Bowman's capsule [26]. In addition, several nicely designed experiments support that hyperplastic epithelial cells are originated from PECs. The cFSGS mice model, established by cell cycle inhibitor p21 knockout, manifested progressive loss of podocytes with collapsed capillary loop and hyperplastic epithelial cells which were negative for WT-1 and nestin (both podocyte specific markers) but positive for PECs markers [27]. Podocyte lineage tracing by genetic tagging in p21 knockout mice proved that proliferating cells within Bowman's space were not podocyte-derived whereas they basically expressed PECs markers [27]. This study strongly suggests that,in cFSGS, proliferating epithelial cells originate from PECs rather than from hyperplastic podocytes* de novo* expressing the PECs phenotype. Dysregulated mitosis and cell cycle may account for the pathogenesis of cFSGS.

In other variants of FSGS the initial pathologic step is regularly the adhesion formation between the glomerular capillary tuft and Bowman's capsule caused by the synechia of the denuded glomerular basement membrane (GBM) to the PECs [28]. The naked GBM is the consequence of the impairment or loss of podocytes. Nonetheless, the role of PECs in the above process is not completely understood. It is not clear if the migration of the activated PECs along the glomerular tuft is limited to the region where podocytes have already been lost or if the invasion of PECs is essentially involved in the injury and loss of podocytes or both. In FSGS patients and three different animal models (5/6-nephrectomy plus DOCA-salt, the transgenic chronic Thy1.1 mice, and the MWF rat), Moeller et al. [17] found that the primary insult which triggered FSGS was associated with PEC activation and cellular adhesions to the capillary tuft. In more detail, activated PECs invaded the engaged capillary tuft and deposited extracellular matrix, and then podocytes were lost and mesangial sclerosis developed. Activated PECs were observed on the tuft where the podocyte marker remained. Therefore, it proposed that activated PECs may impair podocytes and thereby contribute to the initiation and progression of the disease, not just an innocent victim.

#### 2.2.2. PEC Activation in Diabetic Nephropathy (DN)

Activated PECs can sometimes be observed in patients with DN, especially in advanced stage, in our own opinion and also as reported by several other investigators [29, 30]. Occasionally, the proliferation of PECs is prominent with pseudocrescent formation.

In BTBR^ob/ob^ diabetic mice, replacement of leptin restored the number and density of podocytes, accompanied with evident proliferation of PECs. Concomitantly, proteinuria and other morphologic abnormalities were significantly reversed. The authors also proposed that glomerular PECs could function as a progenitor cell niche for podocytes and under proper settings could proliferate and transdifferentiate into podocytes, which may be a pivotal factor for the regression of DN [31]. In another DN animal model, activated and proliferating PECs were observed and associated with overexpressed kidney injury molecule 1 which positively correlated with the extent of proteinuria and podocytopenia in diabetes [32]. Therefore, the pathophysiologic effect of activated PECs on DN remains controversial, probably dependent on the setting of the experiments.

The mechanism of PEC activation in DN is unclear. To our understanding, the endothelium is severely injured in the late phase of diabetic status, which leads to leakage of plasma and consequently induces PEC activation and pseudocrescent formation, partially resembling the mechanisms which account for cellular crescents development in inflammatory CGN.

#### 2.2.3. PEC Activation in Other Glomerular Diseases

Occasionally PEC activation and pseudocrescents formation are presented in membranous nephropathy, progressive glomerulosclerosis, and thrombotic microangiopathy, among others [33, 34]. The mechanisms are not known currently; the activation of PEC may similarly be triggered by the injury of podocyte or endothelium and the seepage of plasma.

## 3. Signaling Pathways Involved in PEC Activation

The signaling pathways that mediate PEC activation are only partially comprehended; however, more and more related observations are emerging. Getting a closer look at them will offer us practical clues for clinical treatment. We summarize the major signaling pathways related to PECs activation in Table 2.

### 3.1. Notch Signaling Pathway in PEC Activation

Notch is a single-transmembrane protein. The family of Notch receptors is evolutionarily conserved through worms to humans [35]. Mammals have four types of Notch receptors (Notch1-4) and five identified Notch ligands (Delta-like 1, 3, and 4 and Jagged 1 and 2). Each of them shows a cell and tissue-specific expression. Upon ligand binding a cascade of proteolytic cleavage events of the Notch receptor is initiated. Firstly it is lysed by disintegrin and metalloproteinase and then cleaved by the *γ*-secretase complex. The final cleavage product, the Notch intracellular domain, a transcription factor, binds to other transcriptional factors and induces the synthesis of Notch target genes such as* Hes* and* Hey* [36–38].

Notch signaling controls cell differentiation in diverse organ systems and also during kidney development. It is detected transiently in prospected podocytes and PECs and is essential for glomerulogenesis [39, 40]. Once glomerular development is completed, Notch activity is significantly decreased; upregulated glomerular Notch activity has been identified in patients with HIV nephropathy, FSGS, systemic lupus erythematous, and diabetes. These findings were also identified in animal models with different kinds of glomerular diseases [41, 42].

In rat PAN nephropathy increased Notch1 expression in podocytes was associated with apoptosis and proteinuria [43]. Nonetheless, in cFSGS transgenic mouse model, upregulated Notch1 was predominant in PECs accompanied by its apoptosis. On the other hand, this finding was not observed in podocytes [44]. Inhibition of Notch signaling markedly reduced PEC hyperplasia in cFSGS mice; conversely, proteinuria and renal morphologic alteration obviously deteriorated which underlines the potential beneficial effects of PEC activation in the setting of advanced podocyte loss. It is well known that the Notch pathway plays pivotal roles in cell migration and phenotypic transformation. For example, inhibition Notch pathway delayed wound healing by preventing cell migration in a skin scratch-scarring model [45]. Similarly, in cultured PECs, Notch inhibition suppressed its migration and mesenchymal phenotypic transition which suggests that Notch-mediated mesenchymal phenotypic alteration and cell migration may compensate for the loss of podocytes.

In addition, a strong upregulation of Notch3 was observed in CD24^+^CD133^+^ PECs in patients with lupus nephritis and FSGS by Lasagni et al. [46]. Blocking the Notch signaling in FSGS model established in SCID mice injected with adriamycin ameliorated proteinuria and prevented podocyte loss in the early stage (7 days) of glomerular insult; however, it hampered CD24^+^CD133^+^ PECs proliferation during the later reparative stage with exacerbating proteinuria and glomerulosclerosis [46]. These observations raise the possibility that the degree of glomerular injury depends on the Notch-mediated balance between podocyte death and renewal offered by PECs.

The trigger of the Notch signaling pathway in PECs in response to podocyte loss requires further investigation although it is known that TGF-*β* is a candidate [44]. Properly temporal and spatial modulation of Notch expression may provide an ideal strategy for glomerular diseases.

### 3.2. Wnt/*β*-Catenin Signaling Pathway in PEC Activation

Wnt/*β*-catenin signaling is an evolutionarily conserved and a multifunctional pathway that regulates cell proliferation and differentiation, angiogenesis, inflammation, and fibrosis. The canonical Wnt pathway through a complicated cascade of reactions prevents transcription factor *β*-catenin degradation and promotes its translocation and accumulation in the nucleus. In the nucleus, *β*-catenin modulates the transcription of Wnt target genes, including genes encoding cyclin D1, VEGF, c-Myc, and CTGF [47, 48].

In kidney organogenesis, the Wnt/*β*-catenin signaling controls both nephrogenesis and ureteric bud development [49, 50]. The Wnt pathway is also involved in the pathogenesis of renal cell carcinoma and Wilms' tumor [51, 52]. The connection of the Wnt signaling pathway with glomerular diseases, cystic kidney diseases, acute renal failure, renal fibrosis, and kidney cancers has been identified and highly concerned [53, 54].

The canonical Wnt/*β*-catenin signaling pathway is specifically involved in podocyte abnormality and proteinuria. Conditional depletion of the *β*-catenin gene in podocytes or the pharmacological inhibition of *β*-catenin by paricalcitol protected mice against proteinuria after adriamycin injection [55, 56]. On the other hand, activation of *β*-catenin by lithium chloride induced proteinuria in mice [55].

As to PECs, Wnt/*β*-catenin activity is indispensable for its lineage specification during the late stages of nephrogenesis which was demonstrated in conditional *β*-catenin knockout mice [57]. Recently it was recognized that developmental pathways are reactivated in injured glomeruli, including the Wnt pathway which plays pivotal roles in the regeneration and repair process. Susztak group found that the Wnt/*β*-catenin pathway modulated podocyte versus PEC marker expression. Increased Wnt/*β*-catenin signaling resulted in the loss of podocyte differentiation markers and the upregulation of PECs specific markers, whereas deletion of *β*-catenin promoted the expression of podocyte markers podocin and WT1 [58, 59]. Therefore Wnt/*β*-catenin signaling likely implicates the transition from PECs to podocytes; however, further convincing evidences are required.

### 3.3. HB-EGF/EGFR Signaling Pathway in PEC Activation

PECs and podocytes* de novo* express heparin-binding epidermal growth factor-like growth factor (HB-EGF) exclusively in human CGN and to a less extent in cFSGS or within the synechia lesion of FSGS [60, 61]. In contrast normal glomerular PECs do not express HB-EGF and this abnormality is not identified in other types of glomerulopathies. One of the receptors for HB-EGF, the EGF receptor (EGFR), is also expressed by PECs and podocytes. In a mouse model of CGN, HB-EGF deficiency and genetic deletion of the HB-EGF alleles or tetracycline-inducible conditional depletion of the EGFR gene in podocytes significantly attenuated CGN development and improved survival rate [62]. This finding indicates that the HB-EGF/EGFR pathway plays an indispensable role in the induction and progression of PEC activation and crescents formation. Targeting HB-EGF/EGFR signaling pathway is a very promising therapeutic approach for CGN.

### 3.4. CXCR4/SDF-1 Signaling Pathway in PEC Activation

CD133^+^CD24^+^ PECs in normal human kidney have been proved to express CXCR4 although it is scarce. However, in patients with CGN the expression of CXCR4 was dramatically enhanced, particularly in the hyperplastic lesions comprised predominantly of PECs. CXCR4 overexpression in PECs was accompanied by upregulation of its ligand, stromal cell-derived factor 1 (SDF-1), in podocytes. In contrast, membranous nephropathy and DN patients with PECs proliferation show only very weak CXCR4 expression [63, 64]. It seems increased CXCR4 expression in PECs is exclusive in proliferative and inflammatory glomerular diseases.

Consistently, Ding et al. [65] reported that the CXCR4/SDF-1 axis was implicated in the interaction and activation of PECs and podocytes in the CGN mice model which is established by von Hippel-Lindaugene deletion in podocytes.

In CGN, the immune complex activates the humoral and cellular immune systems and recruits phlogogenic neutrophils and monocytes/macrophages to the glomerular tuft [66, 67]. The phlogogenic neutrophils can also be primed by anti-neutrophil cytoplasmic antibodies. The inflammatory cells infiltrating the capillary tuft release soluble cytokines and chemokines that penetrate into Bowman's space, ultimately triggering the expression of adhesion molecules and chemokine receptors such as CXCR4 on PECs [68, 69].

### 3.5. Ang II/AT1 Receptor Pathway in PEC Activation

Local production of angiotensin II (Ang II), the main component of the renin-angiotensin system, is upregulated in glomerular disease with proteinuria [70]. Inflammatory cells can release enzymes that produce Ang II, including angiotensin-converting enzyme (ACE) from monocytes/macrophages [71, 72] and cathepsin G in neutrophils [73]. Activation of angiotensin II type 1 (AT1) receptor by Ang II generates cytokines, chemokines, reactive oxygen species, and adhesion molecules which further maintain the inflammatory state.

Activation of the Ang II/AT1 receptor pathway plays a role in cell proliferation and migration [74]. Several studies indicated that inhibition of the ACE could reduce glomerular lesions by limiting PEC migration. Benigni et al. [75] reported that ACE inhibitor reduced the extent of crescents and glomerulosclerosis in MWF rats. The underlying mechanism was that use of the ACE inhibitor resulted in an upregulation of the activity of the cell cycle inhibitor, C/EBP*δ*, thereby preventing PEC proliferation. Accordingly, mitotic activity of cultured PECs was triggered by angiotensin II through blocking of C/EBP*δ*.

In addition, AT1 receptor blocker (ARB) significantly attenuated Ang II initiated PECs proliferation and collagen secretion in vitro. Furthermore combination treatment with ARB and CCR2 antagonist improved renal function in an anti-GBM nephritis model [76].

In human proliferative CGN, Rizzo and coworkers showed that abundant PECs expressed AT1 receptors in patients with proliferative disorders. By contrast, rare PECs detected AT1 receptor immunoreactivity in patients with membranous and diabetic glomerulopathy. Similar to CXCR4/SDF-1 axis, Ang II/AT1 receptor pathway seems only involved in inflammatory crescents formation. [64].

Therefore, blocking the Ang II/AT1 receptor pathways is a highly prospective treatment in patients with proliferative CGN.

### 3.6. Other Pathways Leading to PEC Activation

Several other signaling pathways have also been described for PEC activation, such as the PDGF/PDGFR, LAT/mTORC1, and SSeCKS/cyclin D1 signaling pathways.

In the glomerulus, there is a frequent presence of the PDGF-receptor on the apical and lateral surface of PECs. Van Roeyen et al. [77] found that overexpression of PDGF-D in podocytes induced progressive crescentic glomerulonephritis and also glomerular sclerosis. Thus it is reasonable to speculate that proliferation of PECs and cellular crescents formation result from activation of PDGF-receptor by podocyte originated PDGF-D.

Recently Kurayama et al. [78] showed that amino acid transporter 2 (LAT2) played a critical role in the pathogenesis of CGN by stimulating the mTORC1 signaling pathway in PECs. Treatment with an mTORC1 inhibitor, everolimus, prevented cellular proliferation and maintained the integrity of glomeruli; however, early treatment with everolimus resulted in more fibrinoid necrosis which may be linked to the disruption of protein synthesis through the mTORC1 pathway. Thus, it is speculated that PEC activation may be one of the protective phenomena to overcome glomerular insult. Further investigations are needed to elucidate the time and dose-dependent effects of the mTORC1 inhibitor, a potential candidate for CGN therapy, on PEC activation and crescent development.

In normal glomeruli, Src-suppressed protein kinase C substrate (SSeCKS) is exclusively expressed in PECs and mesangial cells, but not in podocyte [79]. It can sequester cyclin D1 in the cytoplasm in an inactivated status. Once SSeCKS is phosphorylated by aPKC it releases its scaffolding function and consequently induces cyclin D1 translocation from the cytoplasm to nucleus [80, 81]. SSeCKS knockout mice showed hyperplasia of PECs and increased nuclear cyclin D1 expression. These observations suggest that SSeCKS/cyclin D1 pathway affects the mitotic and proliferative properties of PECs [82], and regulating SSeCKS/cyclin D1 signaling may effectively correct the abnormal PECs activation.

## 4. Perspectives

PEC activation is manifested in both CGN and noninflammatory glomerular diseases; nonetheless, the underlying etiology and pathogenesis are variable. This is also true for the signaling pathways involved in PEC activation under different circumstances.

Although, currently, more and more evidences of PEC activation and its underlying signaling events are emerging, what we should pay attention to is that the majority of the related data come from experimental overexpression or knockdown studies, which do not exactly mirror physiological conditions in humans. Also in some experimental settings, PECs are only indirectly affected or associated with the hemodynamic or the paracrine effects. Further studies and understanding of the specific correlation between various signaling pathways and the diverse causes of PEC activation are highly required.

## Figures and Tables

**Table 1:** Glomerular parietal epithelial cells activation in glomerular disease.

	Reference	
	Human	Animal model
CGN	[14]	[9, 14, 19]
FSGS	[14, 17, 26]	[17, 27]
DN	[29, 30]	[31, 32]

CGN: crescentic glomerulonephritis; FSGS: focal segmental glomerulosclerosis; DN: diabetic nephropathy.

**Table 2:** Signaling pathways involved in glomerular parietal epithelial cells activation.

	Human	Animal model
Notch	FSGS, DN [46]	Collapsing FSGS transgenic mice model [44], FSGS SCID mice model [46]
Wnt/*β*-catenin	N/A	Conditional *β*-catenin −/− mice [57]
HB-EGF/EGFR	CGN, FSGS [60, 61]	Anti-GBM induced CGN mice model [62]
CXCR4/SDF-1	CGN [63]	MWF rat with CGN [64], VHL −/− mice [65]
Ang II//AT1 receptor	Proliferative CGN [64]	MWF rat with CGN [75]
LAT2	N/A	Anti-GBM induced CGN rat model [78]
SSeCKS/cyclin D	N/A	SSeCKS −/− mice [82]

## References

[1] Ohse T., Pippin J. W., Chang A. M. (2009). The enigmatic parietal epithelial cell is finally getting noticed: a review. *Kidney International*.

[2] Ohse T., Chang A. M., Pippin J. W. (2009). A new function for parietal epithelial cells: a second glomerular barrier. *The American Journal of Physiology—Renal Physiology*.

[3] Yoshida S., Nagase M., Shibata S., Fujita T. (2008). Podocyte injury induced by albumin overload in vivo and in vitro: involvement of TGF-beta and p38 MAPK. *Nephron Experimental Nephrology*.

[4] Yoder B. K. (2007). Role of primary cilia in the pathogenesis of polycystic kidney disease. *Journal of the American Society of Nephrology*.

[5] Shankland S. J., Smeets B., Pippin J. W., Moeller M. J. (2014). The emergence of the glomerular parietal epithelial cell. *Nature Reviews Nephrology*.

[6] Jefferson J. A., Nelson P. J., Najafian B., Shankland S. J. (2011). Podocyte disorders: core curriculum 2011. *American Journal of Kidney Diseases*.

[7] Smeets B., Kuppe C., Sicking E.-M. (2011). Parietal epithelial cells participate in the formation of sclerotic lesions in focal segmental glomerulosclerosis. *Journal of the American Society of Nephrology*.

[8] Bariéty J., Bruneval P. (2006). Activated parietal epithelial cells or dedifferentiated podocytes in FSGS: Can we make the difference?. *Kidney International*.

[9] Smeets B., Moeller M. J. (2012). Parietal epithelial cells and podocytes in glomerular diseases. *Seminars in Nephrology*.

[10] Zhou W., Hildebrandt F. (2012). Inducible podocyte injury and proteinuria in transgenic zebrafish. *Journal of the American Society of Nephrology*.

[11] Zhang J., Hansen K. M., Pippin J. W. (2012). De novo expression of podocyte proteins in parietal epithelial cells in experimental aging nephropathy. *The American Journal of Physiology—renal Physiology*.

[12] Singh S. K., Jeansson M., Quaggin S. E. (2011). New insights into the pathogenesis of cellular crescents. *Current Opinion in Nephrology and Hypertension*.

[13] Morita T., Suzuki Y., Churg J. (1973). Structure and development of the glomerular crescent. *American Journal of Pathology*.

[14] Smeets B., Angelotti M. L., Rizzo P. (2009). Renal progenitor cells contribute to hyperplastic lesions of podocytopathies and crescentic glomerulonephritis. *Journal of the American Society of Nephrology*.

[15] Bariéty J., Bruneval P., Meyrier A., Mandet C., Hill G., Jacquot C. (2005). Podocyte involvement in human immune crescentic glomerulonephritis. *Kidney International*.

[16] Smeets B., Uhlig S., Fuss A. (2009). Tracing the origin of glomerular extracapillary lesions from parietal epithelial cells. *Journal of the American Society of Nephrology*.

[17] Moeller M. J., Soofi A., Hartmann I. (2004). Podocytes populate cellular crescents in a murine model of inflammatory glomerulonephritis. *Journal of the American Society of Nephrology*.

[18] Atkins R. C., Holdsworth S. R., Glasgow E. F., Matthews F. E. (1976). The macrophage in human rapidly progressive glomerulonephritis. *The Lancet*.

[19] Shimizu M., Kondo S., Urushihara M. (2006). Role of integrin-linked kinase in epithelial-mesenchymal transition in crescent formation of experimental glomerulonephritis. *Nephrology Dialysis Transplantation*.

[20] Ryu M., Migliorini A., Miosge N. (2012). Plasma leakage through glomerular basement membrane ruptures triggers the proliferation of parietal epithelial cells and crescent formation in non-inflammatory glomerular injury. *Journal of Pathology*.

[21] Drew A. F., Tucker H. L., Liu H., Witte D. P., Degen J. L., Tipping P. G. (2001). Crescentic glomerulonephritis is diminished in fibrinogen-deficient mice. *American Journal of Physiology–Renal Physiology*.

[22] Sicking E.-M., Fuss A., Uhlig S. (2012). Subtotal ablation of parietal epithelial cells induces crescent formation. *Journal of the American Society of Nephrology*.

[23] Barisoni L., Kriz W., Mundel P., D’Agati V. (1999). The dysregulated podocyte phenotype: a novel concept in the pathogenesis of collapsing idiopathic focal segmental glomerulosclerosis and HIV-associated nephropathy. *Journal of the American Society of Nephrology*.

[24] Conaldi P. G., Bottelli A., Baj A. (2002). Human immunodeficiency virus-1 Tat induces hyperproliferation and dysregulation of renal glomerular epithelial cells. *The American Journal of Pathology*.

[25] Husain M., Gusella G. L., Klotman M. E. (2002). HIV-1 nef induces proliferation and anchorage-independent growth in podocytes. *Journal of the American Society of Nephrology*.

[26] Dijkman H. B. P. M., Weening J. J., Smeets B. (2006). Proliferating cells in HIV and pamidronate-associated collapsing focal segmental glomerulosclerosis are parietal epithelial cells. *Kidney International*.

[27] Suzuki T., Matsusaka T., Nakayama M. (2009). Genetic podocyte lineage reveals progressive podocytopenia with parietal cell hyperplasia in a murine model of cellular/collapsing focal segmental glomerulosclerosis. *The American Journal of Pathology*.

[28] Elger M., Kriz W. (1998). Podocytes and the development of segmental glomerulosclerosis. *Nephrology Dialysis Transplantation*.

[29] Gaut J. P., Hoshi M., Jain S., Liapis H. (2014). Claudin 1 and nephrin label cellular crescents in diabetic glomerulosclerosis. *Human Pathology*.

[30] Otani N., Akimoto T., Yumura W. (2012). Is there a link between diabetic glomerular injury and crescent formation? A case report and literature review. *Diagnostic Pathology*.

[31] Pichaiwong W., Hudkins K. L., Wietecha T. (2013). Reversibility of structural and functional damage in a model of advanced diabetic nephropathy. *Journal of the American Society of Nephrology*.

[32] Zhao X., Zhang Y., Li L. (2011). Glomerular expression of kidney injury molecule-1 and podocytopenia in diabetic glomerulopathy. *The American Journal of Nephrology*.

[33] Barrett C. M., Troxell M. L., Larsen C. P. (2014). Membranous glomerulonephritis with crescents. *International Urology and Nephrology*.

[34] Ohse T., Vaughan M. R., Kopp J. B. (2010). De novo expression of podocyte proteins in parietal epithelial cells during experimental glomerular disease. *American Journal of Physiology: Renal Physiology*.

[35] Greenwald I. (1998). LIN-12/Notch signaling: lessons from worms and flies. *Genes & Development*.

[36] Ilagan M. X. G., Kopan R. (2007). SnapShot: notch signaling pthway. *Cell*.

[37] Tien A.-C., Rajan A., Bellen H. J. (2009). A notch updated. *Journal of Cell Biology*.

[38] Sharma S., Sirin Y., Susztak K. (2011). The story of Notch and chronic kidney disease. *Current Opinion in Nephrology and Hypertension*.

[39] Bonegio R. G. B., Beck L. H., Kahlon R. K., Lu W., Salant D. J. (2011). The fate of Notch-deficient nephrogenic progenitor cells during metanephric kidney development. *Kidney International*.

[40] Cheng H.-T., Miner J. H., Lin M., Tansey M. G., Roth K., Kopan R. (2003). *γ*-secretase activity is dispensable for mesenchyme-to-epithelium transition but required for podocyte and proximal tubule formation in developing mouse kidney. *Development*.

[41] Sharma M., Callen S., Zhang D., Singhal P. C., Vanden Heuvel G. B., Buch S. (2010). Activation of Notch signaling pathway in HIV-associated nephropathy. *AIDS*.

[42] Murea M., Park J. K., Sharma S. (2010). Expression of notch pathway proteins correlates with albuminuria, glomerulosclerosis, and renal function. *Kidney International*.

[43] Niranjan T., Bielesz B., Gruenwald A. (2008). The Notch pathway in podocytes plays a role in the development of glomerular disease. *Nature Medicine*.

[44] Ueno T., Kobayashi N., Nakayama M. (2013). Aberrant Notch1-dependent effects on glomerular parietal epithelial cells promotes collapsing focal segmental glomerulosclerosis with progressive podocyte loss. *Kidney International*.

[45] Chigurupati S., Arumugam T. V., Son T. G. (2007). Involvement of notch signalling in wound healing. *PLoS ONE*.

[46] Lasagni L., Ballerini L., Angelotti M. L. (2010). Notch activation differentially regulates renal progenitors proliferation and differentiation toward the podocyte lineage in glomerular disorders. *Stem Cells*.

[47] Clevers H. (2006). Wnt/*β*-catenin signaling in development and disease. *Cell*.

[48] Angers S., Moon R. T. (2009). Proximal events in Wnt signal transduction. *Nature Reviews Molecular Cell Biology*.

[49] Merkel C. E., Karner C. M., Carroll T. J. (2007). Molecular regulation of kidney development: is the answer blowing in the Wnt?. *Pediatric Nephrology*.

[50] Lin Y., Liu A., Zhang S. (2001). Induction of ureter branching as a response to Wnt-2b signaling during early kidney organogenesis. *Developmental Dynamics*.

[51] Sadler G. J., Anderson M. R., Moss M. S., Wilson P. G. (2007). Metastases from renal cell carcinoma presenting as gastrointestinal bleeding: two case reports and a review of the literature. *BMC Gastroenterology*.

[52] Lowe L. H., Isuani B. H., Heller R. M. (2000). Pediatric renal masses: wilms tumor and beyond. *Radiographics*.

[53] Kawakami T., Ren S., Duffield J. S. (2013). Wnt signalling in kidney diseases: dual roles in renal injury and repair. *Journal of Pathology*.

[54] Pulkkinen K., Murugan S., Vainio S. (2008). Wnt signaling in kidney development and disease. *Organogenesis*.

[55] Dai C., Stolz D. B., Kiss L. P., Monga S. P., Holzman L. B., Liu Y. (2009). Wnt/*β*-catenin signaling promotes podocyte dysfunction and albuminuria. *Journal of the American Society of Nephrology*.

[56] He W., Kang Y. S., Dai C., Liu Y. (2011). Blockade of Wnt/*β*-catenin signaling by paricalcitol ameliorates proteinuria and kidney injury. *Journal of the American Society of Nephrology*.

[57] Grouls S., Iglesias D. M., Wentzensen N. (2012). Lineage specification of parietal epithelial cells requires *β*-catenin/Wnt signaling. *Journal of the American Society of Nephrology*.

[58] Kato H., Susztak K. (2012). Repair problems in podocytes: wnt, notch, and glomerulosclerosis. *Seminars in Nephrology*.

[59] Kato H., Gruenwald A., Suh J. H. (2011). Wnt/*β*-catenin pathway in podocytes integrates cell adhesion, differentiation, and survival. *The Journal of Biological Chemistry*.

[60] Zeng F., Singh A. B., Harris R. C. (2009). The role of the EGF family of ligands and receptors in renal development, physiology and pathophysiology. *Experimental Cell Research*.

[61] Flamant M., Bollée G., Hénique C., Tharaux P.-L. (2012). Epidermal growth factor: a new therapeutic target in glomerular disease. *Nephrology Dialysis Transplantation*.

[62] Bollée G., Flamant M., Schordan S. (2011). Epidermal growth factor receptor promotes glomerular injury and renal failure in rapidly progressive crescentic glomerulonephritis. *Nature Medicine*.

[63] Mazzinghi B., Ronconi E., Lazzeri E. (2008). Essential but differential role for CXCR4 and CXCR7 in the therapeutic homing of human renal progenitor cells. *Journal of Experimental Medicine*.

[64] Rizzo P., Perico N., Gagliardini E. (2013). Nature and mediators of parietal epithelial cell activation in glomerulonephritides of human and rat. *American Journal of Pathology*.

[65] Ding M., Cui S., Li C. (2006). Loss of the tumor suppressor Vhlh leads to upregulation of Cxcr4 and rapidly progressive glomerulonephritis in mice. *Nature Medicine*.

[66] Sitprija V., Pipatanagul V., Mertowidjojo K., Boonpucknavig V., Boonpucknavig S. (1980). Pathogenesis of renal disease in leptospirosis: clinical and experimental studies. *Kidney International*.

[67] Lai K. N., Aarons I., Woodroffe A. J., Clarkson A. R. (1982). Renal lesions in leptospirosis. *Australian and New Zealand Journal of Medicine*.

[68] Ophascharoensuk V., Pippin J. W., Gordon K. L., Shankland S. J., Couser W. G., Johnson R. J. (1998). Role of intrinsic renal cells versus infiltrating cells in glomerular crescent formation. *Kidney International*.

[69] Lan H. Y., Nikolic-Paterson D. J., Atkins R. C. (1992). Involvement of activated periglomerular leukocytes in the rupture of Bowman's capsule and glomerular crescent progression in experimental glomerulonephritis. *Laboratory Investigation*.

[70] Kinoshita Y., Kondo S., Urushihara M. (2011). Angiotensin II type i receptor blockade suppresses glomerular renin-angiotensin system activation, oxidative stress, and progressive glomerular injury in rat anti-glomerular basement membrane glomerulonephritis. *Translational Research*.

[71] Nahmod K. A., Vermeulen M. E., Raiden S. (2003). Control of dendritic cell differentiation by angiotensin II. *The FASEB Journal*.

[72] Wassmann S., Laufs U., Bäumer A. T. (2001). Inhibition of geranylgeranylation reduces angiotensin II-mediated free radical production in vascular smooth muscle cells: involvement of angiotensin AT1 receptor expression and Rac1 GTPase. *Molecular Pharmacology*.

[73] Sadoshima J. (2000). Cytokine actions of angiotensin II. *Circulation Research*.

[74] McKinney C. A., Fattah C., Loughrey C. M., Milligan G., Nicklin S. A. (2014). Angiotensin-(1-7) and angiotensin-(1-9): function in cardiac and vascular remodelling. *Clinical Science*.

[75] Benigni A., Morigi M., Rizzo P. (2011). Inhibiting angiotensin-converting enzyme promotes renal repair by limiting progenitor cell proliferation and restoring the glomerular architecture. *American Journal of Pathology*.

[76] Urushihara M., Ohashi N., Miyata K., Satou R., Acres O. W., Kobori H. (2011). Addition of angiotensin II type 1 receptor blocker to CCR2 antagonist markedly attenuates crescentic glomerulonephritis. *Hypertension*.

[77] Van Roeyen C. R. C., Eitner F., Boor P. (2011). Induction of progressive glomerulonephritis by podocyte-specific overexpression of platelet-derived growth factor-D. *Kidney International*.

[78] Kurayama R., Ito N., Nishibori Y. (2011). Role of amino acid transporter LAT2 in the activation of mTORC1 pathway and the pathogenesis of crescentic glomerulonephritis. *Laboratory Investigation*.

[79] Nelson P. J., Moissoglu K., Vargas J., Klotman P. E., Gelman I. H. (1999). Involvement of the protein kinase C substrate, SSeCKS, in the actin-based stellate morphology of mesangial cells. *Journal of Cell Science*.

[80] Lin X., Nelson P., Gelman I. H. (2000). SSeCKS, a major protein kinase C substrate with tumor suppressor activity, regulates G_1_→S progression by controlling the expression and cellular compartmentalization of cyclin D. *Molecular and Cellular Biology*.

[81] Lin X., Gelman I. H. (2002). Calmodulin and cyclin D anchoring sites on the Src-suppressed C kinase substrate, SSeCKS. *Biochemical and Biophysical Research Communications*.

[82] Burnworth B., Pippin J., Karna P. (2012). SSeCKS sequesters cyclin D1 in glomerular parietal epithelial cells and influences proliferative injury in the glomerulus. *Laboratory Investigation*.

